# History of Mosquitoborne Diseases in the United States and Implications for New Pathogens

**DOI:** 10.3201/eid2405.171609

**Published:** 2018-05

**Authors:** Max J. Moreno-Madriñán, Michael Turell

**Affiliations:** Fairbanks School of Public Health, Indiana University–Purdue University Indianapolis, Indianapolis, Indiana, USA (M.J. Moreno-Madriñán);; VectorID LLC, Frederick, Maryland, USA (M. Turell)

**Keywords:** socioeconomic conditions, climate, vector-borne infections, mosquitoborne diseases, anthroponoses, zoonoses, yellow fever, malaria, West Nile, dengue, Zika, viruses, United States

## Abstract

The introduction and spread of West Nile virus and the recent introduction of chikungunya and Zika viruses into the Americas have raised concern about the potential for various tropical pathogens to become established in North America. A historical analysis of yellow fever and malaria incidences in the United States suggests that it is not merely a temperate climate that keeps these pathogens from becoming established. Instead, socioeconomic changes are the most likely explanation for why these pathogens essentially disappeared from the United States yet remain a problem in tropical areas. In contrast to these anthroponotic pathogens that require humans in their transmission cycle, zoonotic pathogens are only slightly affected by socioeconomic factors, which is why West Nile virus became established in North America. In light of increasing globalization, we need to be concerned about the introduction of pathogens such as Rift Valley fever, Japanese encephalitis, and Venezuelan equine encephalitis viruses.

The recent explosive outbreaks of disease throughout the Americas caused by the introduction of Zika and chikungunya viruses has raised several questions, including whether these or similar disease-causing pathogens could spread into countries located at temperate latitudes, particularly into the continental United States. The conventional perception is that diseases caused by mosquito-transmitted pathogens are mostly associated with tropical areas (*1*). Indeed, such areas include the ranges of temperature and other climatic conditions that are ideal for the vectors of these pathogens. Moreover, because such diseases are clearly more prevalent in these areas, we might easily assume that the association between the tropics and mosquito-transmitted pathogens indicates that temperate regions are at less risk for these diseases because of their cooler climates. However, this expectation of safety should not be taken for granted. In fact, cases of disease caused by mosquito-transmitted pathogens such as West Nile virus (WNV) occur readily in North America (*2*), and several encephalitides occasionally occur in the United States (*3*). In addition, history reveals that yellow fever and malaria were once very common in the United States and resulted in millions of cases (*4*,*5*). More than 100,000 deaths occurred in the United States in the 18th and 19th centuries from yellow fever alone (*4*) (Figure), in areas considered as temperate or cold according to the Köppen-Geiger climate classification scheme (*6*).

**Figure:**
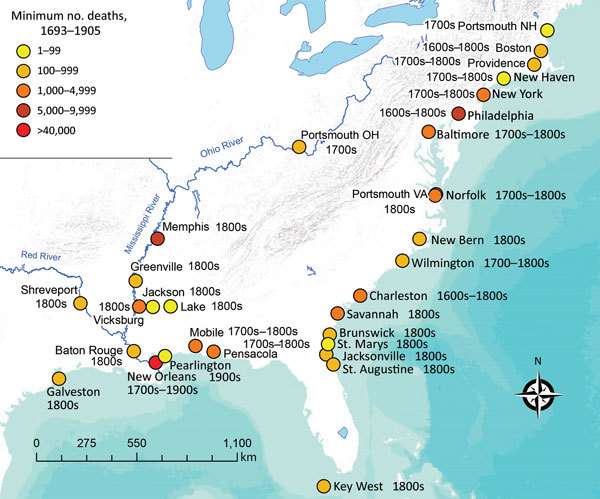
Outbreaks of yellow fever reported during 1693–1905 among cities comprising part of present-day United States. Because deaths from yellow fever were often not recorded, or only referred to as “a significant portion of the population” and thus not accounted for here, the numbers presented in this figure represent a minimum for the cities cited (*4*,*6*–*8*).

## Historical Aspects

The *Aedes aegypti* mosquito and 2 of its transmitted viruses (yellow fever and dengue viruses) and the plasmodia parasites of malaria (*Plasmodium vivax* and *P. falciparum*) are believed to have arrived to the Americas during the 17th century by ship during the slave trade (*7*–*9*). During the 17th century (even during a cold period known as the Little Ice Age) until the 19th century, summertime malaria was present in much of the eastern United States, including northern areas of the country (*10*). Numerous outbreaks of malaria occurred as far north as Massachusetts, with documented outbreaks occurring during 1793–1799 and in 1806, 1810, 1820, 1828, and 1836; nearly 2,000 cases were reported during an outbreak in 1880 alone (*11*). In the subtropical southern states along the Mississippi Valley during the 18th and 19th centuries, malaria spread quickly, especially during the American Revolutionary War and Civil War (5). Malaria is reported to have caused ≈1,300,000 cases of illness and ≈10,000 deaths among soldiers during the 4 years of the Civil War (*5*). Although mosquito transmission of disease-causing organisms was not proved until 1889, the coincidental use of window and door screens might have contributed to the lowering prevalence by the beginning of the 1880s (*12*).

Efforts to control mosquitoes by draining mosquito larval habitat sites have been undertaken since 1930 by the US government (*13*). However, such efforts might have been improperly managed, and the depopulation of the rural South likely was the main factor leading to the substantial reduction in malaria by the early 1940s (*13*). In 1947, the National Malaria Eradication Program, a cooperation between the state and local health agencies of 13 southeastern states and the Communicable Disease Center of the US Public Health Service, commenced the operations that presumably resulted in the elimination of malaria from the United States (*14*). The application of DDT and drainage of mosquito larval habitats were the main control methods used. By the early 1950s, after 3 years with no indigenous cases (based on eradication criteria stated by the National Malaria Society at the time), malaria was considered to be no longer endemic in any given area of the continental United States (*14*). Although 63 outbreaks of locally transmitted mosquitoborne malaria were reported in the United States during 1957–2003, these outbreaks consisted of only 156 cases (*15*) and were caused by mosquitoes infected after biting persons that had acquired the pathogen in other countries (*16*). Still, concern about malaria exists because the 3 mosquito species that transmitted malaria in the United States before its eradication, *Anopheles quadrimaculatus*, *An. freeborni*, and *An. pseudopunctipennis*, along with other anopheline species, are still present in the country (*16*). Thus, rather than the mere control of the vector, the introduction of screening, air conditioning, television, and other enhancements (i.e., improved socioeconomic conditions), along with improved early diagnosis and access to treatment, might also have helped eliminate human reservoirs and eradicate malaria from the United States.

Yellow fever epidemics were common in northeastern US cities as far north as Boston, Massachusetts; Portsmouth, New Hampshire; and Providence, Rhode Island, during the 18th century and the beginning of the 19th century (*4*,*17*). The large epidemic of yellow fever in Philadelphia, Pennsylvania, in the summer of 1793 (resulting in ≈5,000 deaths) was a contributing factor in the decision to move the nation’s capital to the city of Washington (*18*), and epidemics in 1798 in Philadelphia and New York, New York, resulted in 3,500 and 2,080 deaths, respectively (*4*). After 1822, yellow fever epidemics in the United States were generally restricted to more southern cities (*4*). In addition, major epidemics occurred in New Orleans, Louisiana, in 1853 (≈9,000 deaths) (*4*) and Savannah, Georgia, in 1876 (1,066 deaths) (*4*). In the great epidemic of 1878, 16,000–20,000 deaths from yellow fever occurred along the Mississippi River, from the Gulf of Mexico to Memphis, Tennessee, and St. Louis, Missouri (*4*). Overall, at least 100,000 deaths were attributed to yellow fever in the United States during 1693–1905 (*4*). Within a few years after the discovery in 1901 that yellow fever was transmitted by mosquito, the last locally acquired cases of yellow fever in the United States were reported in 1905 in Pearlington, Mississippi (46 deaths), and New Orleans (437 deaths) (*4*,*11*). It is important to note that open cisterns were common in homes of the Mississippi Gulf Coast from the 18th century through the early 20th century and that ≈5,000 deaths from yellow fever occurred in Mississippi roughly within the same period (*11*). Important aspects in commonly characterizing these yellow fever epidemics were the tendency for them to occur during summer and fall and in port cities with active trade with the Caribbean Islands (Figure). Today, these aspects still represent a concern but now are not just limited to port cities as our increasingly global society experiences increasing numbers of travelers flying from tropical areas (*19*), especially during the summer season.

On the basis of identification of *Ae. aegypti* mosquitoes as the primary vector of yellow fever by the Walter Reed Commission in 1901 and the earlier discovery by Ronald Ross that the parasite that causes malaria is transmitted by mosquito (*20*), the first actions taken by US companies that took over the construction of the Panama Canal in Panama was the eradication of mosquitoes, mainly by draining or oiling of larval rearing sites and fumigation with pyrethrum (*21*). In 1947, the Pan American Sanitary Bureau, later renamed the Pan American Health Organization, implemented a program that lasted until 1970 to eradicate *Ae. aegypti* mosquitoes and consequently dengue (*7*). The mosquito was declared eradicated from 18 continental countries in the Americas in 1962 (*22*). The apparent success of the elimination program, along with environmental concerns about the use of pesticides such as DDT, were among the reasons to end it. However, since 1971, *Ae. aegypti* mosquitoes have started regaining their previous geographic distribution, including a notable increase occurring after 2000 (*7*). The failure to eradicate this vector in the Americas was a major factor leading to the reemergence of dengue (*22*) and very likely to the recent appearance of chikungunya and Zika viruses. Dengue outbreaks have continued to occur in southern Texas and southern Florida as recently as 2011 (*23*,*24*). However, these outbreaks have been more associated with the high proportion of persons traveling from dengue-endemic countries than to climatic or socioeconomic conditions.

## Socioeconomic Changes and Anthroponotic Pathogens

As we have mentioned, the mosquito vectors capable of transmitting malaria, yellow fever, and dengue have been present throughout much of the United States since the 1600s (*4*,*7*,*11*). What has clearly changed in the United States from the 18th and 19th centuries to the present is the availability of potable water, sanitation, and social lifestyles. These developments have essentially eliminated the need to store water in indoor containers and reduced contact with mosquitoes. After World War II, and particularly during the 1950s, a boom in the US economy increased the standard of living and aided the widespread use of television and air conditioning. In addition, the use of screened terraces and windows increased. These commodities influenced persons to spend a longer time indoors or in screened areas (thus decreasing outdoor exposure to mosquito bites) and made indoor environments less accessible to outside mosquitoes. Because changes in living conditions in the United States reduced the opportunity for contact between mosquitoes and humans, these changes substantially affected the transmission of those pathogens for which humans are the primary amplifying host. Such pathogens fall into the group of anthroponoses (i.e., those in which humans are the principal vertebrate host). Thus, diseases such as malaria, yellow fever, and dengue have all but disappeared (*4*,*7*,*9*,*14*–*16*), and the viruses that cause chikungunya and Zika, which arrived into the United States after living conditions had improved, have not become established in the continental United States despite the occurrence of ≈10,000 imported cases of these infections since 2014 (*25*,*26*).

The impact of changing cultural and socioeconomic conditions on the prevalence of anthroponotic diseases over time has also been observed when the cultural and socioeconomic conditions change across geographic areas. Contemporary observations of dengue prevalence in contiguous cities at the United States–Mexico border indicate lower dengue prevalence and higher socioeconomic conditions on the US side (*27*,*28*). The lower rates of dengue, chikungunya, and Zika virus infection reported in the United States compared with Latin America countries coincide with higher socioeconomic conditions in the United States (*29*). Although malaria and yellow fever are transmitted by different mosquitoes and the larval habitats and biting behaviors of these mosquitoes are different, both diseases are similar in that humans are essentially the only amplifying vertebrate host. Therefore, continued transmission would require considerable mosquito–human contact, and either a reduction in the number of mosquitoes present or their ability to bite humans would be necessary to reduce the potential for continued transmission.

## Potential for the Introduction of Zoonotic Viruses

This association between socioeconomic status and prevalence of diseases caused by mosquito-transmitted pathogens applies more to anthroponotic than to zoonotic pathogens (i.e., those pathogens in which animals other than humans play a major role in the transmission cycle). These zoonotic viruses cause diseases such as WNV infection, St. Louis encephalitis, eastern equine encephalitis, western equine encephalitis, and La Crosse encephalitis. These viruses are maintained in natural transmission cycles involving various mosquito and bird or rodent species and therefore are not usually greatly affected by improved housing for humans. Because humans are not involved in the transmission cycle of these viruses, the viruses persist in the United States, whereas anthroponotic pathogens (e.g., malaria and dengue and yellow fever viruses), which involve only humans as vertebrate amplifying hosts, have been essentially eliminated (*4*,*7*,*8*,*14*–*16*). In contrast, zoonotic viruses, such as eastern equine encephalitis and La Crosse viruses, continue to be a cause of disease (*30*,*31*), and the introduction of WNV in 1999 (*32*) illustrates the potential for an exotic virus to become established in North America. Since its introduction in 1999, WNV has spread throughout the continental United States as well as to southern Canada and most of Central and South America (*33*,*34*). In the continental United States, ≈40,000 cases and ≈2,000 deaths have been attributed to WNV infection (*2*). Thus, unlike the anthroponotic viruses chikungunya and Zika, which failed to become established in the continental United States despite the introduction of ≈10,000 imported cases (*25*,*26*), exotic zoonotic viruses could very likely be introduced and become established, with potentially devastating consequences.

## Recent History of the Introduction of Novel Viruses

Like yellow fever and dengue viruses, chikungunya and Zika viruses are also anthroponotic viruses transmitted by *Ae. aegypti* mosquitoes*.* These viruses were recently introduced into the Americas, where each has caused massive outbreaks in South and Central America as well as in the Caribbean, resulting in ≈2 million infections with chikungunya virus (*35*) and ≈750,000 infections with Zika virus (*36*). These outbreaks have led to the introduction of numerous imported cases into the continental United States, where ≈3,500 imported cases of chikungunya virus infection (*25*) and ≈5,000 imported cases of Zika virus infection (*26*) have been reported to the Centers for Disease Control and Prevention (CDC). In addition to the *Ae. aegypti* mosquito*,* the *Ae. albopictus* mosquito has been implicated as a potential vector of Zika (*37*,*38*) and chikungunya (*39*,*40*) viruses. The first locally acquired chikungunya case in the continental United States was reported in Florida in July 2014 (*41*). As of December 31, 2016, CDC has reported 3,869 chikungunya cases in the United States, of which 13, all in southern Florida, have been identified as locally transmitted (*25*). Zika virus is of particular concern because of its potential to cause Guillain-Barré syndrome and congenital abnormalities such as microcephaly (*42*,*43*) and because it can be transmitted not only through mosquito bites but also from mother to child, by blood transfusion, and through sexual contact (*44*). Indeed, the first case identified in the United States (in 2008) was attributed to sexual transmission (*45*). As of July 12, 2017, CDC has reported a total of 5,381 cases of Zika virus infection in the continental United States, including 49 cases acquired through sexual transmission or routes other than mosquito transmission and 224 cases assumed to be transmitted locally by mosquito bite (*26*). Local mosquito transmission was first reported in July 2016 in southern Florida (*46*), and genetic studies indicate that 4–40 separate introductions of the virus contributed to the outbreaks (*47*). In addition, similar to dengue and chikungunya, cases of locally transmitted Zika virus have been identified in southern Texas (*48*). Most of these introductions originated from the Caribbean, as indicated by genetic and surveillance analysis and supported by the high influx of travelers from this high-incidence region (*47*). As we previously mentioned, the active trade between the Caribbean and the United States played a key role in influencing the abundance of yellow fever outbreaks in the United States during 1693–1905 (*4*). This history demonstrates not only the potential threat for the arrival of new pathogens from tropical origins but, in general, the risk of globalization for the introduction of new pathogens as a whole and the importance of paying attention to the public health of developing countries as a way to ensure the safety of local public health everywhere.

## Conclusions

Although anthroponotic pathogens such as yellow fever virus and malaria were once prevalent in the United States (including the northern states), their prevalence occurred when socioeconomic conditions were poor. However, when these conditions improved, such diseases virtually disappeared. Since 2014, despite the introduction of ≈10,000 imported cases of Zika or chikungunya virus infections, relatively few local cases have been reported, and all of them occurred in areas where *Ae. aegypti* mosquitoes were present (i.e., neither of these 2 viruses has managed to become established in the continental United States). On the other hand, as illustrated by WNV, a zoonotic virus has the potential to be introduced and become established. Zika and chikungunya viruses failed to become established because they are anthroponotic viruses, whereas WNV was able to become established because it is a zoonotic virus (some other zoonotic viruses such as Japanese encephalitis and Rift Valley fever viruses show similar potential). Improvement in standards of living inhibits anthroponotic but not zoonotic viruses. Given that the decreased exposure of humans to mosquitoes in the United States is primarily driven by changes in socioeconomic conditions, it is important to note that these very conditions could be threatened by massive natural disasters or any other similarly disruptive event. Consequently, appropriate disaster preparedness plans need to be in place to address this potential threat.
